# Design and Construction of Cu(OH)_2_/Ni_3_S_2_ Composite Electrode on Cu Foam by Two-Step Electrodeposition

**DOI:** 10.3390/mi13020237

**Published:** 2022-01-30

**Authors:** Sa Lv, Wenshi Shang, Huan Wang, Xuefeng Chu, Yaodan Chi, Chao Wang, Jia Yang, Peiyu Geng, Xiaotian Yang

**Affiliations:** Key Laboratory for Comprehensive Energy Saving of Cold Regions Architecture of Ministry of Education, Jilin Provincial Key Laboratory of Architectural Electricity & Comprehensive Energy Saving, Jilin Jianzhu University, Changchun 130118, China; shangws0426@163.com (W.S.); wanghuan@jlju.edu.cn (H.W.); stone2009@126.com (X.C.); chiyaodan@jlju.edu.cn (Y.C.); wangchao@jlju.edu.cn (C.W.); yangjia@jlju.edu.cn (J.Y.); gengpeiyu1@163.com (P.G.)

**Keywords:** electrode material, Cu(OH)_2_, Ni_3_S_2_, Cu foam, electrodeposition

## Abstract

A Cu(OH)_2_/Ni_3_S_2_ composite has been designed and in situ constructed on Cu foam substrate by facile two-step electrodeposition. Cu(OH)_2_ is achieved on Cu foam by galvanostatic electrodeposition, and the subsequent coating of Ni_3_S_2_ is realized by cyclic voltammetric (CV) electrodeposition. The introduction of Cu(OH)_2_ provides skeleton support and a large specific surface area for the Ni_3_S_2_ electrodeposition. Benefiting from the selection of different components and preparation technology, the Cu(OH)_2_/Ni_3_S_2_ composite exhibits enhanced electrochemical properties with a high specific capacitance of 4.85 F cm^−2^ at 2 mA cm^−2^ and long-term cyclic stability at 80.84% (4000 cycles).

## 1. Introduction

Recently, transition metal sulfides—especially nickel sulfide—such as NiS, Ni_3_S_2_, Ni_9_S_8_ have attracted extensive attention as a promising cathode for supercapacitors [1,2,3]. Compared with their corresponding oxides and hydroxides, transition metal sulfides have better conductivity and electrochemical activity due to the lower electronegativity of sulfur [4,5]. However, these available nickel-sulfide-based electrode materials still have problems, such as low-rate capability and restricted electrochemical redox reaction kinetics at large current density. Many works have focused on constructing nickel sulfide-based composite to solve these problems, including hybridizing with high conductive agents such as carbon fiber and graphene, to increase the conductivity and compounding with other components to give full play to the synergy of each component [6,7,8]. In addition, researchers also designed the composite components from the perspective of regulating the material structure and growth process. For example, Xing et al. designed ZnO@Ni_3_S_2_ array on Ni foam with a high specific capacitance of 1529 F g^−^^1^ at 2 A g^−^^1^ [9]. The enhanced capacitance is ascribed to backbone support of ZnO array, which provides a large specific surface area for subsequent growth of Ni_3_S_2_, and the uniform core/shell structure with good stability and facilitates the charges transport during the processes of charging and discharging. At the same time, ZnO was chosen because of its mature synthetic pathway, which facilitates the regulation of diverse structures and morphologies [10]. Taking a similar view, other ZnO-based materials, including ZnO@Co(OH)_2_ [11], ZnO/Ni(OH)_2_ [12], ZnO/CoS [13], and ZnO@CoFe_2_O_4_ [14] have been explored. Although enhanced capacitance could be achieved through assistance with ZnO, ZnO has almost no capacitance characteristics, and it may partly dissolve in a long-term strong acidic or alkaline test environment. The most typical candidate with similar advantages to ZnO is Cu(OH)_2_. An especially prominent advantage of Cu(OH)_2_ is that Cu foam can act as both electrode substrate and the only Cu source to achieve the in situ growth of Cu(OH)_2_, and the characteristic of this in situ growth is to avoid impurities, increase yield and reduce contact resistance. Moreover, Cu(OH)_2_ can be calcined to obtain CuO, which can also be used as an active electrode component. In general, there are two ways to achieve the in situ growth of Cu(OH)_2_ on Cu foam substrate. One is in situ oxidation treatment; that is, the Cu foam is oxidized by a mixture of (NH_4_)_2_S_2_O_8_ and NaOH aqueous solutions at room temperature [15,16]. The other is an electrodeposition technique, and the essence of the reaction is in situ electrochemical anodic oxidation [17,18]. The latter has the advantage of simple operation, a short experimental period and strong repeatability. In terms of the electrodeposition device, it usually adopts a two-electrode or three-electrode system. Compared with a three-electrode system, the two-electrode system has fewer influencing factors because no reference electrode is involved, and thus it is bound to have higher repeatability.

Based on the above analysis, herein, a two-electrode system was adopted to achieve in situ growth of Cu(OH)_2_ nanostructures on Cu foam substrate followed by coating of Ni_3_S_2_ through subsequent CV electrodeposition. Cu(OH)_2_, as an effective active component, participates in the electrochemical reaction, and provides a large specific surface area for the growth of Ni_3_S_2_. Compared with the single-component Ni_3_S_2_, the electrochemical properties of Cu(OH)_2_/Ni_3_S_2_ composite are significantly improved.

## 2. Materials and Methods

### 2.1. Materials

Cu foam (70 PPI, 1.8 mm thick, Kunshan DESSCO Co., Ltd., Kunshan, China) was cut into 1 × 1.5 cm^2^ slices, and then the slices were washed in dilute hydrochloric acid, acetone, ethanol and deionized water to remove the surface oxide layer. The chemical reagents used in the experiment included nickel chloride hexahydrate (NiCl_2_·6H_2_O), thiourea (CH_4_N_2_S), KOH and NaOH.

### 2.2. Preparation of Cu(OH)_2_/Ni_3_S_2_ Electrode

In the first step, a two-electrode setup was exploited to electrodeposit Cu(OH)_2_ in 2 M KOH solution. Cu foam and Pt plate acted as the working and counter electrode, respectively. The electrochemical oxidation was carried out at a current density of 0.05 A cm^−2^ for 20 min.

In the second step, a three-electrode setup was adopted to achieve Ni_3_S_2_ electrodeposition. The Cu(OH)_2_ obtained in the previous step served as working electrode, and saturated calomel electrode (SCE) and Pt plate served as reference and counter electrode, respectively. The CV electrodeposition was carried out for 20 segments in the potential window of −1.245 to 0.156 V with a scan rate of 0.005 V s^−1^ in a mixture solution containing 0.48 g of NiCl_2_ and 3.04 g of CH_4_N_2_S.

### 2.3. Characterization

XRD (Cu Kα radiation), FE-SEM (JSM-7610F) and XPS (ESCALAB 250Xi) were used to characterize the structure and morphology of the sample. CHI 760E Electrochemical workstation was used to complete the electrochemical tests, such as cyclic voltammetry (CV), galvanostatic charge–discharge (GCD) and cyclic stability. The test system consists of Cu(OH)_2_/Ni_3_S_2_ composite, Hg/HgO and Pt plate, which served as working, reference and counter electrode, respectively. The electrolyte is 2 M NaOH aqueous solution.

## 3. Results

**Figure 1:**
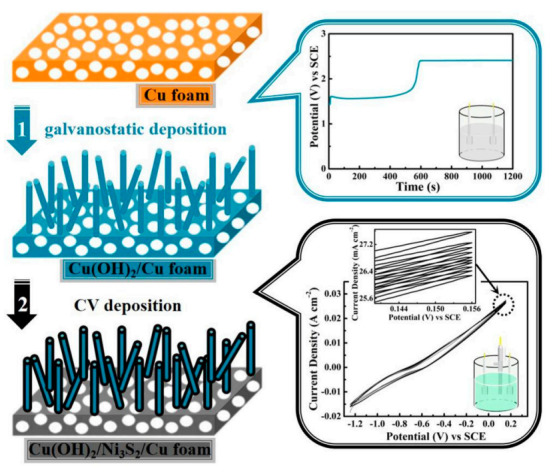
Schematic illustration of the in situ construction process of Cu(OH)_2_/Ni_3_S_2_ composite: (1) galvanostatic deposition; (2) CV deposition.

Figure 1 depicts the Cu(OH)_2_/Ni_3_S_2_ composite constructed by two-step electrodeposition. Homogeneous and dense Cu(OH)_2_ nanorods are firstly deposited on the Cu foam substrate by two-electrode galvanostatic deposition, and the Cu(OH)_2_/Ni_3_S_2_ composite is eventually obtained by the continuous coating of Ni_3_S_2_ on the above Cu(OH)_2_ nanorods surface by CV electrodeposition in a three-electrode system.

**Figure 2:**
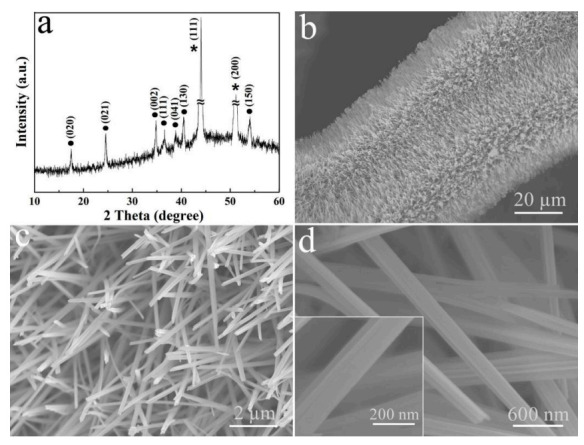
(**a**) XRD pattern and (**b**–**d**) FE-SEM images of Cu(OH)_2_ nanorods formed on Cu foam substrate at different magnifications.

Figure 2a shows the XRD pattern of the sample that is deposited on the Cu foam through the galvanostatic electrodeposition technique. Two strong peaks attributed to Cu foam are labeled with an asterisk (JCPDS card No. 01-1241). The other diffraction peaks, labeled with a dot, are consistent with (020), (021), (002), (111), (041), (130) and (150) planes of orthorhombic Cu(OH)_2_ (JCPDS No.13-0420). The morphology and microstructure of Cu(OH)_2_ are displayed in Figure 2b–d. Figure 2b is a low-magnification FE-SEM image, which demonstrates that the original Cu foam surface (Figure S1) is uniformly covered by filament-like Cu(OH)_2_ with high density. The corresponding enlarged image in Figure 2c confirms that Cu(OH)_2_ nanorods are distributed uniformly on the Cu substrate with smooth surface and a diameter of 150 ± 20 nm (Figure 2d). The inset of Figure 2d gives a typical single Cu(OH)_2_ nanorod, which clearly confirms the above conclusion.

In order to study the growth process of Cu(OH)_2_ on Cu foam substrate, the effects of current density and electrodeposition time on the morphology of Cu(OH)_2_ were investigated in detail. The FE-SEM images of Cu(OH)_2_ obtained at different current density (Figure S2a–c) demonstrate bundles of Cu(OH)_2_ nanorods are scattered on Cu substrate, leaving partially exposed Cu substrate visible when the current density is 0.01 A cm^−2^. As the current density is increased to 0.025 A cm^−2^, the number of Cu(OH)_2_ nanorods increases significantly, and the Cu substrate is covered with dense and uniform Cu(OH)_2_ nanorods (Figure S2d–f). Further doubling of the current density leads to the generation of more dense Cu(OH)_2_ nanorods, as shown in Figure 2b. However, as the current density continues to increase to 0.075 A cm^−2^, the surface of the Cu substrate is damaged by the large current and obvious cracks are formed (Figure S2g,f). The FE-SEM images of Cu(OH)_2_ obtained with different electrodeposition time show only a small amount of Cu(OH)_2_ nanorods with sharp tips emerging from the Cu substrate at the early stage of electrodeposition (1 min, Figure S3a,b). Upon increasing the electrodeposition time to 5 min, as seen in Figure S3c,d, the Cu substrate is completely covered with Cu(OH)_2_ nanorods, and the nanorods continue generating when the electrodeposition reaches 20 min (Figure 2b). However, when the electrodeposition time is prolonged to 1 h (Figure S3e,f), these Cu(OH)_2_ nanorods collapse and crush each other as they grow longer.

**Figure 3:**
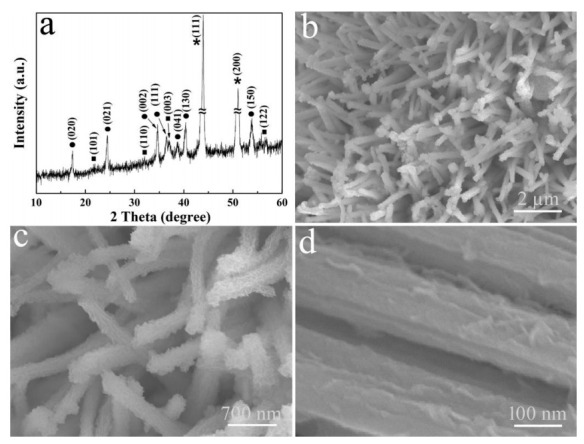
(**a**) XRD pattern and (**b**–**d**) FE-SEM images of Cu(OH)_2_/Ni_3_S_2_ composite at different magnifications.

The Cu(OH)_2_/Ni_3_S_2_ composite was further achieved after subsequent CV electrodeposition. The XRD pattern of the sample (Figure 3a) presents three types of diffraction peaks. Except that the peaks labeled with asterisks and dots are derived from the Cu foam and Cu(OH)_2_, respectively, the peaks labeled with squares are ascribed to Ni_3_S_2_ obtained by (JCPDS card No. 44-1418). The corresponding FE-SEM image in Figure 3b demonstrates that the nanorod-shape of Cu(OH)_2_ is unchanged, while the surface becomes rough due to the coverage and wrapping of Ni_3_S_2_. Besides, the diameter of these nanorods is increased to 240 ± 30 nm (Figure 3c). The enlarged image in Figure 3d confirms that these Ni_3_S_2_ are bent nanoflakes with a thickness of ca. 50 nm.

**Figure 4:**
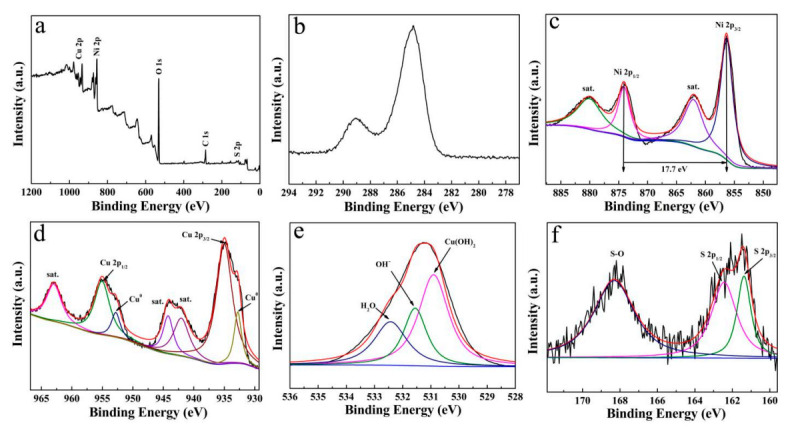
XPS spectra of (**a**) survey scan; (**b**) C 1s; (**c**) Ni 2p; (**d**) Cu 2p; (**e**) O 1s; (**f**) S 2p of Cu(OH)_2_/Ni_3_S_2_ electrode.

The XPS spectra of Cu(OH)_2_/Ni_3_S_2_ composite are presented in Figure 4, which are calibrated with C 1s at 284.8 eV (Figure 4b). The full spectrum in Figure 4a confirms that Cu, Ni, O, C and S are present, and no obvious impurity is detected. The Ni 2p spectrum in Figure 4c mainly contains two peaks at 856.50 and 874.26 eV, corresponding to Ni 2p_3/2_ and Ni 2p_1/2_, respectively. Separation energy of 17.7 eV indicates the existence of Ni^2+^ and Ni^3+^ [19,20]. Both of them have a satellite peak at 861.70 and 880.00 eV. In regard to Cu 2p spectrum (Figure 4d), two main characteristic peaks located at 935.10 and 954.50 eV, with satellite peaks at 962.94, 944.80 and 941.90, correspond to Cu 2p_3/2_ and Cu 2p_1/2_, respectively. The two separated small peaks at 952.56 and 932.82 eV are attributed to the exposed Cu substrate during the test [21]. The O 1s spectrum in Figure 4e can be divided into three peaks with binding energies at 530.90, 531.70 and 532.80 eV, corresponding to Cu(OH)_2_, OH^-^ and H_2_O molecule, respectively [22]. Figure 4f exhibits the S 2p spectrum, and the peaks centered at 162.30 and 161.40 eV belongs to S 2p_1/2_ and S 2p_3/2_, respectively, which can be assigned to S^2-^. In addition, the characteristic peak of S-O at 168.40 eV in the S 2p spectrum indicates the oxygen of the hydroxyl group is bonded to the S bond and adsorbed on the surface of the electrode material [23,24,25]. The hydroxyl group is derived from the hydrolysis of thiourea as follows:

(1) $${\left(NH_{2}\right)}_{2}CS+2H_{2}O↔H_{2}S+CO_{2}+2NH_{3}$$

(2) $$NH_{3}+H_{2}O\to NH_{3}\cdot H_{2}O\to NH^{4+}+OH^{-}$$

**Figure 5:**
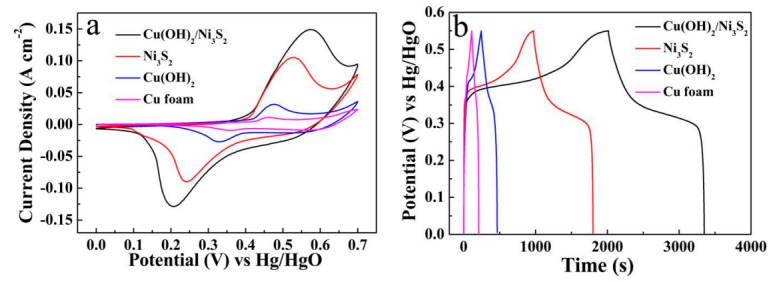
(**a**) CV curves at 10 mV s^−1^ and (**b**) GCD curves at different current densities of Cu(OH)_2_/Ni_3_S_2_, Ni_3_S_2_, Cu(OH)_2_ electrode and Cu foam substrate.

CV and GCD measurements were carried out to explore the electrochemical performances of the Cu(OH)_2_/Ni_3_S_2_ composite. Figure 5a records the CV curves of the four electrodes when the scan rate is set at 10 mV s^−1^, including Cu(OH)_2_/Ni_3_S_2_, Ni_3_S_2_, Cu(OH)_2_ electrodes and Cu foam substrate. Among them, Cu(OH)_2_/Ni_3_S_2_ represents the largest enclosing area, and thus provides the maximum specific capacitance. Meanwhile, the CV curve of Cu(OH)_2_/Ni_3_S_2_ shows a pair of typical redox peaks (0 to 0.7 V), which is attributed to Ni^2+^/Ni^3+^ originating from the Faradaic reactions as Equation (3) [23]. For Cu(OH)_2_ electrode, a pair of redox peak at about 0.46 and 0.32 V can be observed, corresponding to the electrochemical transformation between Cu^2+^ and Cu^+^ as described by Equation (4) [26,27]:

(3) $$Ni_{3}S_{2}+3OH^{-}↔Ni_{3}S_{2}{\left(OH\right)}_{3}+3e^{-}$$

(4) $${2\;\mathrm{Cu}(\mathrm{OH})}_{2}{+2\;e}^{-}\;↔{\;\mathrm{Cu}}_{2}{O+2\;\mathrm{OH}}^{-}{+H}_{2}O$$

**Figure 6:**
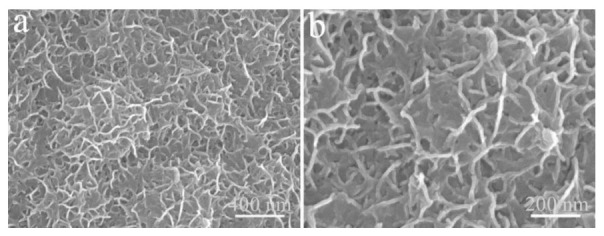
FE-SEM images of single Ni_3_S_2_ electrodeposited on the Cu foam substrate at different magnifications, (**a**) 400 µm; (**b**) 200 nm.

Figure 5b compares the GCD curves of the four electrodes mentioned above at 2 mA cm^−2^. Obviously, the Cu(OH)_2_/Ni_3_S_2_ reveals the longest discharge time, illustrating the largest specific capacitance [22]. The capacitance characteristics of Cu(OH)_2_ and Cu foam are rather weak, while the electrochemical performance of Ni_3_S_2_ is significantly improved by the addition of Cu(OH)_2_. In the absence of Cu(OH)_2_, Ni_3_S_2_ appears as an irregular network structure scattered on the surface of the Cu foam (Figure 6). Therefore, the Cu(OH)_2_ nanorods function as skeletons and provide abundant active sites for the subsequent Ni_3_S_2_ deposition.

**Figure 7:**
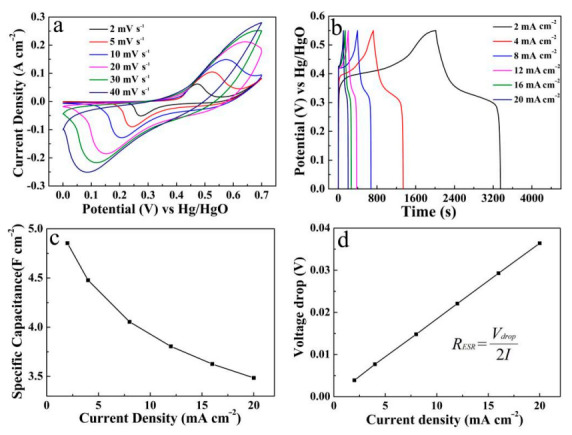
(**a**) CV curves; (**b**) GCD curves; (**c**) line diagram of specific capacitance at different current densities and (**d**) voltage drops of the Cu(OH)_2_/Ni_3_S_2_ composite deposited on Cu foam substrate.

**Figure 8:**
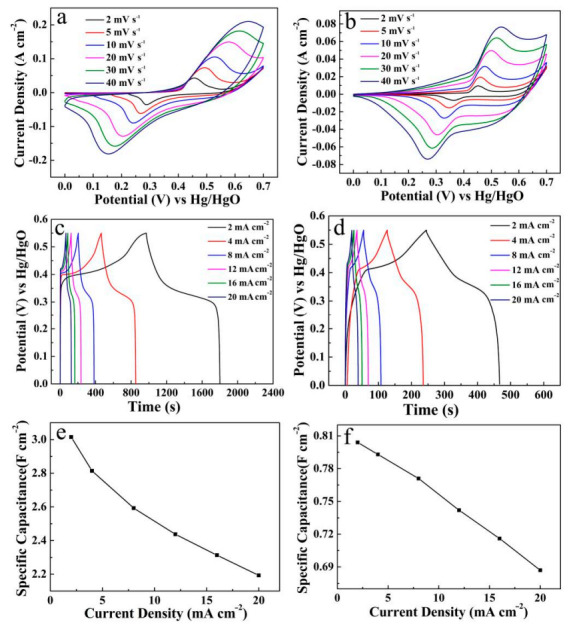
CV curves, GCD curves and line diagram of specific capacitance at different current densities of Ni_3_S_2_ (**a**,**c**,**e**) and Cu(OH)_2_ (**b**,**d**,**f**).

The Cu(OH)_2_/Ni_3_S_2_ composite was further carried out via CV tests at various scan rates (2–40 mV s^−1^), as seen in Figure 7a. The pair of redox peaks exhibits the same variation trend and standard symmetry under the appropriate potential window. It is evident that, at all kinds of scan rates, the large scan rate is bound to a large enclosing area, while the specific capacitance decreases accordingly. This decrease is attributed to the fact that the participation of the internal electrochemical active sites is limited due to the restriction of ions/electrons diffusion at a relatively large scan rate [15]. Figure 7b describes the GCD measurements at different current densities of 2–20 mA cm^−2^. The nonlinearity of the GCD curves reflects the obvious pseudocapacitance characteristic of the electrode material. The specific capacitance value of Cu(OH)_2_/Ni_3_S_2_ electrode can be calculated using the equation in Supplementary Materials [28]. Figure 7c lists the specific capacitance up to 4.85, 4.48, 4.05, 3.81, 3.63 and 3.48 F cm^−2^ corresponding to discharge current densities of 2, 4, 8, 12, 16 and 20 mA cm^−2^, respectively. In addition, the average RESR is calculated to be 0.90 Ω cm^−2^ [27], as shown in Figure 7d. Meanwhile, the CV curves, GCD curves and the corresponding line diagram of specific capacitance at different current densities of single Ni_2_S_3_ and Cu(OH)_2_ were also recorded for comparison in Figure 8.

**Figure 9:**
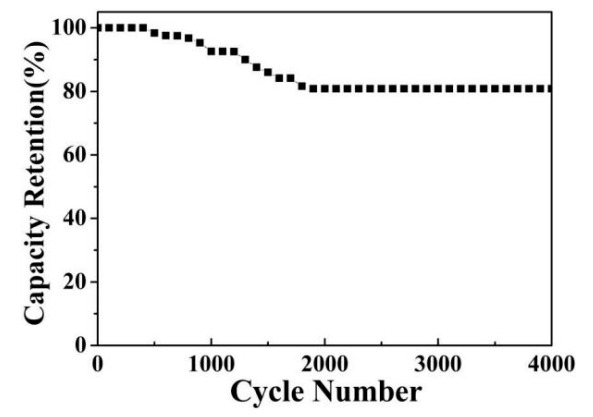
Cyclic stability of the Cu(OH)_2_/Ni_3_S_2_ electrode.

**Figure 10:**
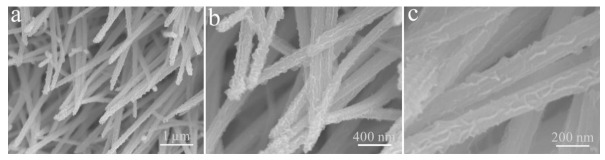
(**a**–**c**) FE-SEM images of the Cu(OH)_2_/Ni_3_S_2_ composite at different magnifications after electrochemical test.

Figure 9 reveals the cyclic stability of the Cu(OH)_2_/Ni_3_S_2_ electrode. The specific capacitance reaches 90.00% of the initial value after 1300 cycles and stabilizes at 80.84% within 4000 cycles. At the same time, the main structure and morphology of the Cu(OH)_2_/Ni_3_S_2_ composite have no obvious change after the cycle test (Figure 10).

Table 1 shows a comparison of the specific capacitance of the Cu(OH)_2_/Ni_3_S_2_ composite in this work with those electrode materials containing Cu(OH)_2_ or Ni_3_S_2_ in the literature. It demonstrates our sample is comparable to or even better than the recently reported electrode materials. The enhanced electrochemical properties of the Cu(OH)_2_/Ni_3_S_2_ composite can be attributed to both the composition of the material and the preparation technique. In terms of components, the selection of Cu(OH)_2_ has three advantages: (1) Cu foam serves as both a current collector and the only Cu source to achieve the in situ growth of Cu(OH)_2_. The in situ growth strategy reduces the contact resistance and facilitates the transmission of ions/electrons. At the same time, the products can be directly used as a binder-free electrode for an electrochemical performance test [27]; (2) the generated rod-shaped Cu(OH)_2_ provides skeleton support and large specific surface area for the subsequent Ni_3_S_2_ electrodeposition [26]; (3) Cu(OH)_2_, also exhibits some pseudocapacitance characteristics as an effective active component [15]. In terms of preparation technology, the in situ electrodeposition technique is easy to manipulate and has a short experimental period. More importantly, the electrode to be measured can be obtained directly [17].

## 4. Conclusions

The Cu(OH)_2_/Ni_3_S_2_ electrode has been designed and constructed on Cu foam substrate by two-step electrodeposition. The combination of the composition of the material and preparation technology advantages endows the Cu(OH)_2_/Ni_3_S_2_ electrode superior electrochemical performance with a high specific capacitance of 4.85 F cm^−2^ at 2 mA cm^−2^ and long-term cyclic stability at 80.84% after 4000 cycles. This facile method provides an effective route to prepare other Cu(OH)_2_ and CuO-based composite electrode materials.

## Figures and Tables

**Table 1:** A comparison of various Cu(OH)_2_ or Ni_3_S_2_ containing electrode materials.

Specific Capacitance	Electrode Substrate	Electrolyte	Current Density	Specific Capacitance	Refs.
Cu(OH)_2_	Cu foam	5 M NaOH	2 mA cm^−^^2^	2.15 F cm^−^^2^	[15]
Cu(OH)_2_	carbon cloth	1 M NaOH	1 mA cm^−^^2^	0.24 F cm^−^^2^	[29]
Ni_3_S_2_	Ni foam	2 M KOH	1 mA cm^−^^2^	2.52 F cm^−^^2^	[30]
Co(OH)_2_/CoOOH/ Co_3_O_4_/Cu(OH)_2_	Cu foam	1 M KOH	1 mA cm^−^^2^	1.94 F cm^−^^2^	[31]
Cu(OH)_2_@MnO_2_	Cu foam	6 M KOH	2 mA cm^−^^2^	0.71 F cm^−^^2^	[26]
C/NiMn-LDH/Ni_3_S_2_	Ni foam	3 M KOH	2 mA cm^−^^2^	3.49 F cm^−^^2^	[7]
Co_3_S_4_-Ni_3_S_2_	Ni foam	6 M KOH	2 mA cm^−^^2^	2.83 F cm^−^^2^	[8]
Ni_3_S_2_@ppy	Ni foam	2 M KOH	2 mA cm^−^^2^	3.15 F cm^−^^2^	[32]
Ni_3_S_2_/rGO	Ni foam	2 M KOH	2 mA cm^−^^2^	1.96 F cm^−^^2^	[33]
Cu(OH)_2_/Ni_3_S_2_	Cu foam	2 M NaOH	2 mA cm^−^^2^	4.85 F cm^−^^2^	this work

## Supplementary Materials

The following are available online at https://www.mdpi.com/article/10.3390/mi13020237/s1, Figure S1: FE-SEM image of pristine Cu foam; Figure S2: FE-SEM images of Cu(OH)_2_ formed on Cu foam substrate at different current densities: (a–c) 0.01 A cm^−2^; (d–f) 0.025 A cm^−2^; (g,h) 0.075 A cm^−2^. Figure S3: FE-SEM images of Cu(OH)_2_ formed on Cu foam substrate at different electrodeposition time: (a,b) 1 min; (c,d) 5 min; (e,f) 1 h. Equations for calculating specific capacitance.
