# Supplementary material for: Design and Construction of Cu(OH)2/Ni3S2 Composite Electrode on Cu Foam by Two-Step Electrodeposition
Source: Micromachines (Basel). 2022 Jan 30;13(2):237. doi: 10.3390/mi13020237 (PMC8878843; doi:10.3390/mi13020237)
Supplement: Supplementary file 1 [file micromachines-13-00237-s001.zip › micromachines-1532739-supplementary.pdf]

Supplementary Materials

Design and Construction of $Cu(OH)_2/Ni_3S_2$ Composite Electrode on Cu Foam by Two-Step Electrodeposition

Sa Lv *, Wenshi Shang, Huan Wang, Xuefeng Chu, Yaodan Chi, Chao Wang, Jia Yang, Peiyu Geng and Xiaotian Yang *

Key Laboratory for Comprehensive Energy Saving of Cold Regions Architecture of Ministry of Education, Jilin Provincial Key Laboratory of Architectural Electricity & Comprehensive Energy Saving, Jilin Jianzhu University, Changchun 130118, China; shangws0426@163.com; wanghuan@jlju.edu.cn; stone2009@126.com; chiyaodan@jlju.edu.cn; wangchao@jlju.edu.cn; yangjia@jlju.edu.cn; gengpeiyu1@163.com

* Correspondence: lvsa82@163.com (S.L.); hanyxt@163.com (X.Y.); Tel.: +86-0431-8456-6181 (S.L.)

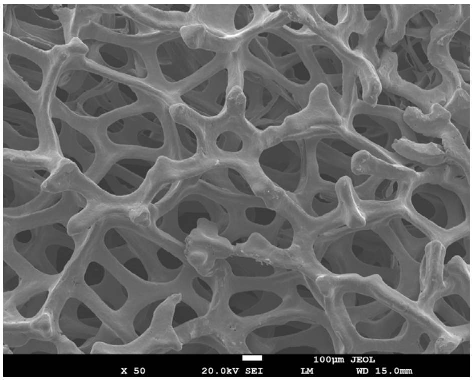

Figure S1. FE-SEM image of pristine Cu foam.

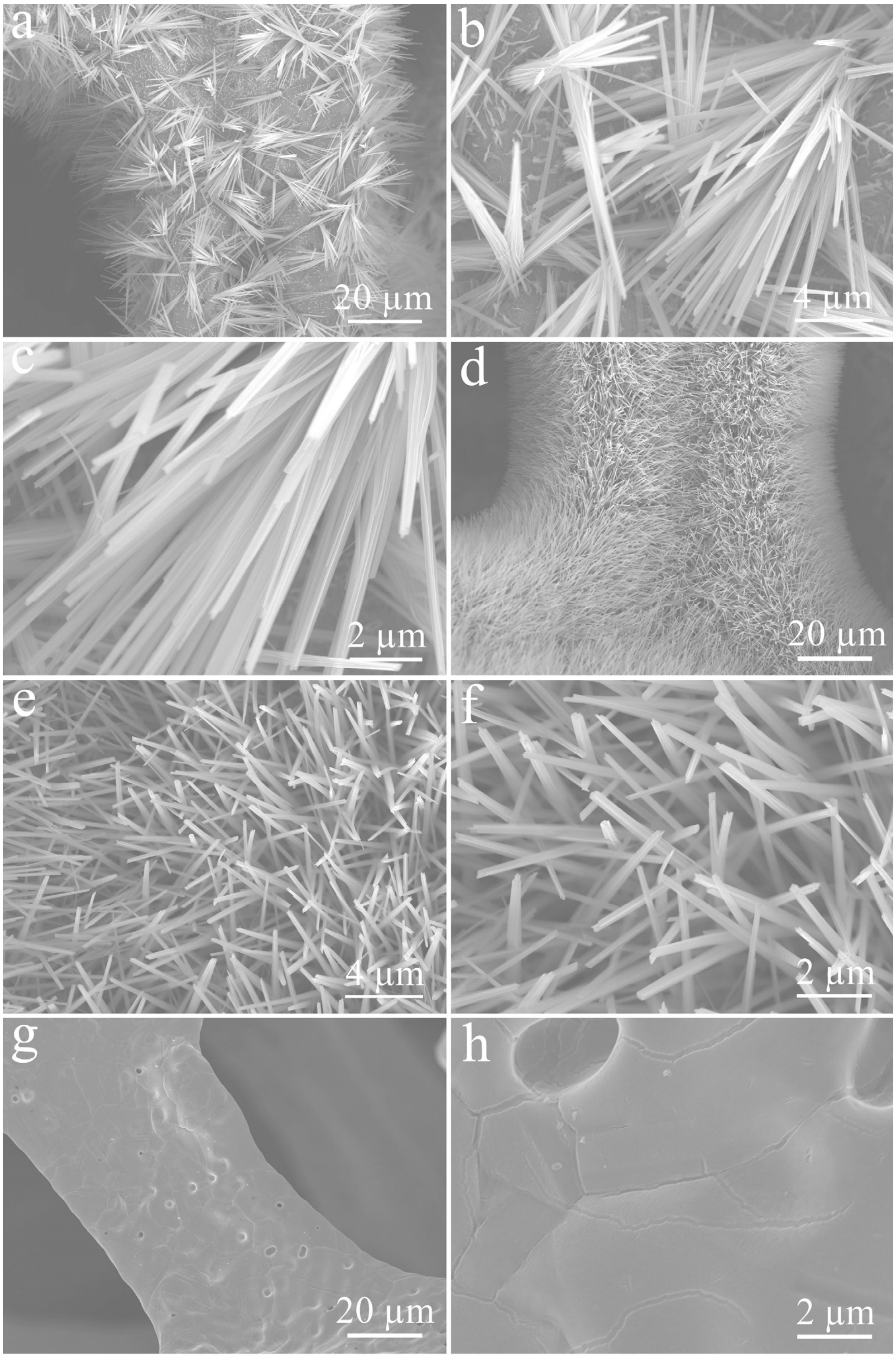

Figure S2. FE-SEM images of $Cu(OH)_2$ formed on Cu foam substrate at different current densities: **(a-c)** 0.01 A cm^{-2} ; **(d-f)** 0.025 A cm^{-2} ; **(g-h)** 0.075 A cm^{-2}.

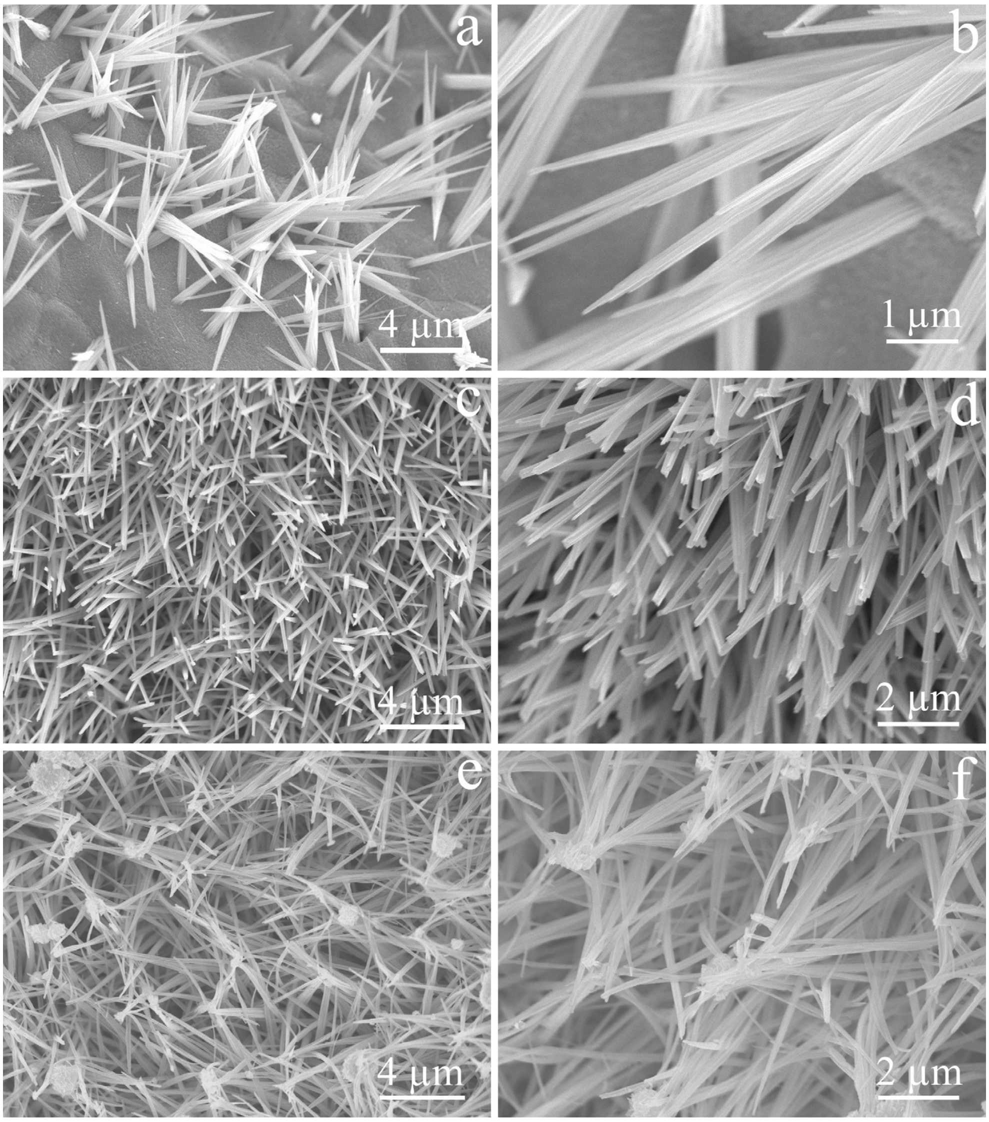

Figure S3. FE-SEM images of $Cu(OH)_2$ formed on Cu foam substrate at different electrodeposition time: **(a-b)** 1 min ; **(c-d)** 5 min; **(e-f)** 1 h.

The areal capacitance of $Cu(OH)_2/Ni_3S_2$ electrode is calculated according to the equation:

$$C_S = \frac{I \times \Delta t}{S \Delta V}$$

where C_s (F cm^{-2}) is the specific capacitance, I (A) is the charge–discharge current, Δt (s) is the discharging time, S (cm^2) is the effective area of the electrode and ΔV (V) represents the potential drop during discharge.
